# Sex-Specific Patterns of Force Plate-Derived Predictors for Vertical Jump Performance and Algorithmic Musculoskeletal Injury Risk in College Athletes

**DOI:** 10.3390/sports14020067

**Published:** 2026-02-05

**Authors:** Adam Eckart, Pragya Sharma Ghimire

**Affiliations:** Department of Health and Human Performance, Kean University, 1000 Morris Avenue, Union Township, NJ 07083, USA; pghimire@kean.edu

**Keywords:** force plates, countermovement vertical jump, injury risk

## Abstract

Background: Force plate-derived metrics are increasingly used to assess performance and monitor musculoskeletal injury risk, yet the mechanisms linking jump-mechanics patterns to injury risk remain unclear, particularly when using proprietary, algorithmically derived risk scores. Clarifying these relationships is important for improving screening practices, program design, and load management in athletic populations. Methods: A total of 233 collegiate athletes completed countermovement vertical jump (CMVJ) testing on a commercial force plate, which produced 26 force-time variables and proprietary composite metrics. LASSO regression with bootstrapping identified important predictors of CMVJ height and algorithmically derived musculoskeletal injury (AMSKI risk), and Partial Least Squares (PLS) models characterized multivariate patterns across force-time variables. Sex-stratified analyses and post hoc modeling examined potential mechanisms. Results: Greater AMSKI risk was associated with a coordinated pattern of greater concentric output, including greater power, velocity, and impulse, combined with reduced braking capacity. Braking rate of force development (“Load”) showed an inverse association with AMSKI risk across sexes, and females in the elevated-risk category displayed significantly reduced braking values. Postural control measures contributed differently by sex. PLS models indicated that both CMVJ height and AMSKI risk reflected interactions among multiple variables, while proprietary composite scores showed inconsistent alignment with mechanistic predictors. Conclusions: Multivariate force-time profiling offers practical value for identifying athletes whose high-output movement strategies may elevate injury risk when braking control is insufficient. Because proprietary, algorithmically derived risk metrics show inconsistent associations with underlying mechanics, further independent validation is needed before such scores are used in clinical or training decisions.

## 1. Introduction

Musculoskeletal injuries are very prevalent among college athletes [1]. Over time, research has demonstrated the reliability and validity of the countermovement vertical jump (CMVJ) in assessing lower-body performance and is routinely used by strength and conditioning coaches, as well as sports scientists [2]. Vertical jumping assessment is an essential aspect of athletic performance and is linked to success in sports involving lower-body explosiveness, such as basketball, volleyball, and football. It is also associated with performance factors, such as strength, speed, and agility [3]. CMVJ performance can be measured using various tools, including contact mats, force plate platforms, accelerometers, high-speed cameras, and infrared platforms, all of which have been shown to provide valid and reliable measures of CMVJ performance [2].

Recent technological advances have led to the development of commercially available force plates that are increasingly used and studied in sports and military settings [4]. Commercial force plates that provide force-time curve variables are often used to monitor neuromuscular fatigue and to manage workloads throughout training cycles or sport seasons [5,6]. Force plates are generally considered the gold standard for assessing CMVJ performance, providing essential measures such as the reactive strength index (RSI) or the modified reactive strength index (mRSI), which are associated with lower-body performance and sports injuries [7,8,9]. RSI is a measure used to evaluate an individual’s ability to transition from an eccentric to a concentric contraction rapidly, often used to monitor, assess, and reduce the risk of injury in athletes [10]. Maximal strength, especially relative to body mass, appears to have a strong relationship with RSI, indicating that stronger athletes tend to have better reactive strength [10]. Evidence suggests interlimb RSI asymmetries during the drop jump may better predict future injury than vertical jump height alone [8,9]. Inter-limb asymmetry may increase the risk of injury in sports, as the stronger leg may sustain excessive stress due to greater reliance and loading. Conversely, the weak leg may be compromised to maintain even an average load [11]. Furthermore, asymmetries may result in unequal force absorption or a loss of multi-plane stability, both of which are essential for sustaining the impacting forces [11].

The equipment and procedural demands of the drop jump test may limit its practicality for field-based assessment of jumping and landing function. In contrast, commercial force plates offer a more streamlined approach for evaluating deficits in lower-body function. However, studies utilizing CMVJ on a force plate to assess ground reaction forces in relation to injury are inadequate [8]. Some new force plate systems attempt to quantify injury risk by deriving metrics from jump performances to create composite scores [12]. For example, the Sparta Science force plate platform provides multifaceted, proprietary performance metrics derived from the force-time curve. The Sparta Score is a composite score algorithmically calculated from three metrics and their balance, including “Load” (the average braking rate of force development), “Explode” (the average relative vertical concentric force), and “Drive” (the average relative vertical concentric impulse). Importantly, the Load variable used by Sparta is distinct from training load, which typically quantifies the amount of stress imposed on the body during physical activities. Purportedly, greater Sparta scores indicate a better balance between the component measures [4]. The Sparta force plate also provides an AMSKI risk category based on the musculoskeletal health score, with greater scores indicating reduced injury risk [4].

Given the novelty of this emerging technology and the increasing likelihood of widespread adoption, more research is needed to clarify the relationships between force plate-derived metrics, proprietary injury risk scores, and injury mechanisms, as data-driven insights may offer additional utility for coaches, athletes, and clinicians. Moreover, given the large number of measures generated by commercial force plates such as the Sparta system, systematic methods for filtering and identifying the most relevant predictors of algorithmically derived injury risk and vertical jump performance should be further explored. Therefore, the objectives of this study were to (1) demonstrate methods for filtering force plate measures based on their predictive importance for CMVJ height and AMSKI risk category in male and female athletes; (2) evaluate the contribution of composite scores to the prediction of CMVJ performance and AMSKI risk; (3) analyze patterns in Sparta force plate metrics associated with elevated AMSKI risk and CMVJ height; and (4) examine whether AMSKI risk classifications align with established biomechanical and neuromuscular injury mechanisms.

## 2. Materials and Methods

This secondary analysis of 233 Division III collegiate athletes included male (*n* = 132) and female athletes (*n* = 101) between the ages of 19 and 25 years from 11 different sports who participated in a single testing session. Athletes were included if they were free of injury, had no other contraindications to exercise, and were medically cleared to participate. Athletes with injuries or undergoing rehabilitation at the time of testing were excluded from the study. Each participant performed three CMVJs on the Sparta force plate at a sampling frequency of 1000 Hz, with a minimum of 30 s between trials (Sparta Science, v1.9.31; Sparta Software Corp., San Francisco, CA, USA). The best trial was retained for analysis. All data were de-identified and extracted by the sports medicine team after completion of team physicals. This study was reviewed, deemed exempt under 45 CFR 46.104(d)(4)(ii), and approved by the university’s Institutional Review Board (IRB #24-081506).

### 2.1. Stratification and Data Preprocessing

**Figure 1:**
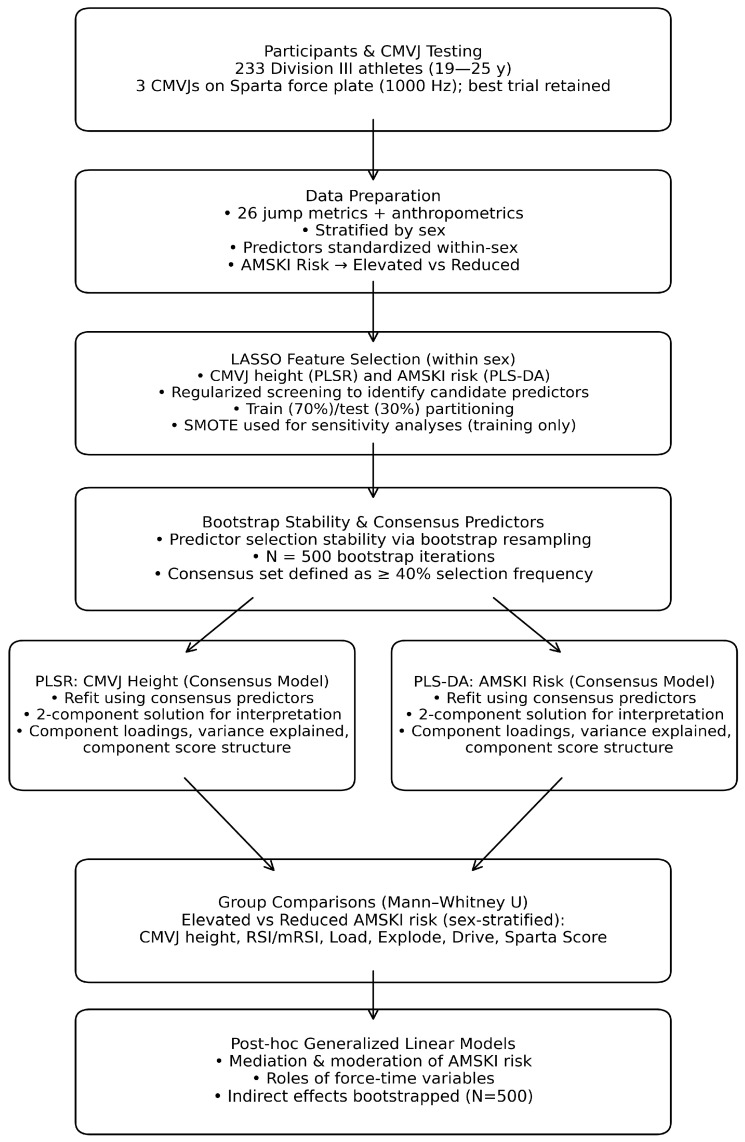
Data processing and analytical workflow.

Figure 1 summarizes the data processing and analytical workflow. Before the analyses, all data were stratified by sex. Normality was checked using the Kolmogorov–Smirnov test (*p* < 0.05). All continuous predictor variables were standardized within-sex using z-scores before the analysis. Differences between Elevated and Reduced AMSKI risk for CMVJ, RSI/mRSI, and across specific force plate-derived measures (Load, Explode, and Drive) were analyzed using the Mann–Whitney U test (*p* < 0.05). Algorithmic injury risk categories were condensed into a dichotomous variable: Elevated Risk and Reduced Risk. “High”, “Average”, and “Above Average” risk categories were grouped as “Elevated Risk,” while “Below Average” and “Low” risk categories were grouped as “Reduced Risk.” Statistical analyses were conducted using IBM SPSS Statistics (Version 30; IBM Corp., Armonk, NY, USA) and Python (Version 3.13.5; Wilmington, DE, USA).

### 2.2. LASSO Feature Selection

To account for strong predictor relationships, robust feature selection was performed using LASSO regression models, which were separately fitted within sex for CMVJ and the elevated AMSKI risk category. The LASSO algorithm was trained and tested on 70% and 30% of the data, respectively. Twenty-six different jump measures, provided as output measures from the Sparta force plate platform, along with height, body mass, body mass index (BMI), and age, were standardized using parameters derived from the training set and then applied to training sample splits and test data. As a sensitivity analysis, bootstrap resampling with and without Synthetic Minority Oversampling Technique (SMOTE) was applied within each bootstrap resample (N = 500) of the training set before fitting the sparse models. LASSO with and without SMOTE was performed using 5-fold cross-validation on a predefined grid of regularization parameters. To evaluate the stability of the predictors retained in the sparse models, bootstrap resampling (N = 500) was performed to determine the selection frequency of variables with non-zero coefficients. Selection frequencies obtained under SMOTE and non-SMOTE conditions were compared to evaluate the robustness of predictor inclusion to class imbalance.

### 2.3. Partial Least Squares Analyses

Due to the number of predictors and the potential for substantial collinearity, pattern analysis for the elevated AMSKI risk category, as determined by the Sparta force plate algorithm, was conducted using Partial Least Squares Discriminant Analysis (PLS-DA) with 5-fold cross-validation. PLS-DA is a supervised learning method that projects samples into a new space defined by latent variables (linear combinations of the original predictors) optimized for class separation. Pattern analysis was also performed for CMVJ height using Partial Least Squares Regression (PLSR) with 5-fold cross-validation. Prior to modeling and cross-validation, the PLS-DA and PLSR models were refit using only the predictors retained in at least 40% of the LASSO bootstrap resamples, yielding consensus predictor sets and loadings for each outcome within sex. Since LASSO is likely to select only one member of a correlated cluster per bootstrap and because many force-time variables are mechanically linked, we used a selection-frequency threshold of 40% to ensure that predictors consistently contributing to the latent structure would be retained.

To quantify uncertainty in the model performance estimates arising from the holdout sample size, test-set performance metrics (area under the receiver operating characteristic curve [AUC], precision–recall curve [positive predictive value {PPV}], accuracy, root mean squared error [RMSE], and coefficient of determination [R^2^]) were bootstrapped (N = 500). For parsimony and interpretability, only the first two PLS components were used for analysis and visualization, as higher-order components were not expected to meaningfully contribute to outcome interpretation. Vector biplots were generated to visualize the relative contributions of each predictor to both CMVJ height and elevated AMSKI risk, as quantified by their consensus loadings from the PLSR and PLS-DA, respectively.

### 2.4. Post Hoc Analyses

Following the primary analyses, generalized linear modeling was conducted to further examine potential mechanisms underlying the relationships among force plate variables and AMSKI risk. Mediation and moderation analyses were performed to test whether the force-time curve variables served compensatory or efficiency-based roles in modulating AMSKI risk. Standardized beta (β) coefficients were interpreted descriptively to characterize the relative strength of associations (β ≈ 0.10–0.29 small, 0.30–0.49 moderate, ≥0.50 large). Indirect effects were estimated using bootstrap resampling (N = 500) with statistical significance set at *p* < 0.05.

## 3. Results

### 3.1. Sex Differences in Algorithmically Derived Injury Risk

A total of 53 (23%) athletes were classified as having elevated AMSKI risk. Of those in the elevated-risk category, 31 were male (58%), and 22 were female (42%). In males, CMVJ, Drive, Explode, RSI, mRSI, and the Sparta Score were significantly increased in the elevated-risk group (*p* < 0.01) (Table 1). Similarly, in females, CMVJ, Drive, RSI, and mRSI were significantly increased in the elevated-risk group (*p* ≤ 0.01). However, Load was significantly reduced in the female elevated-risk group. There were no significant differences for Load in males, or for Explode and the Sparta Score in females.

### 3.2. LASSO-Selected Predictors for AMSKI Risk (PLS-DA Models)

In males, LASSO bootstrap resampling retained 23 consensus predictors in the No-SMOTE condition compared to 26 predictors under SMOTE (Table 2, Figures S1 and S2). While COPy unweighting displacement, RSI, and countermovement depth were retained only in the SMOTE condition, the remaining predictors were retained in both the No-SMOTE and SMOTE conditions. In females, 23 predictors were retained in the No-SMOTE condition compared to 27 predictors under SMOTE (Figures S4 and S5). Most predictors were shared between the No-SMOTE and SMOTE conditions, except for maximum acceleration, unweighting time, COPx sway velocity, and COPy sway velocity, which were retained only in the SMOTE condition.

### 3.3. PLS-DA Consensus Model Performance (AMSKI Risk)

**Figure 2:**
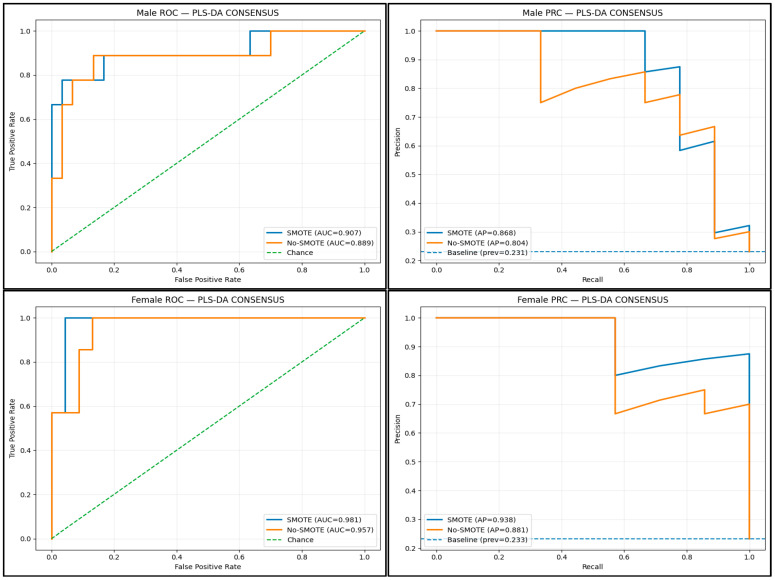
SMOTE and No-SMOTE ROC and PRC curves in males (**top**) and females (**bottom**) for the consensus PLS-DA AMSKI risk models.

ROC curve analysis for the PLS-DA consensus models demonstrated excellent discrimination with and without SMOTE in classifying elevated AMSKI risk. In males, the AUC for the SMOTE and no-SMOTE consensus models were 0.91 and 0.89, respectively (Figure 2, Table S2). In females, the AUC for the SMOTE and no-SMOTE consensus models were 0.98 and 0.96, respectively (Figure 2, Table S2). The SMOTE and no-SMOTE PPVs in males were 0.87 and 0.80, whereas the PPVs in females were 0.84 and 0.88, respectively (Table S2). In males, the No-SMOTE condition achieved higher accuracy (0.90) than the SMOTE condition (0.79) (Table S2). Conversely, in females, accuracy was slightly greater in the SMOTE condition (0.90) than in the No-SMOTE condition (0.87) (Table S2). Across all performance metrics, 95% bootstrap confidence intervals for SMOTE–No-SMOTE differences crossed zero for both males and females, indicating no advantage of synthetic oversampling (Table S4 and Table S5). These findings support the stability of the No-SMOTE models and indicate that classification performance was not driven by resampling strategy. In males and females, respectively, the first two PLS-DA components explained 38% and 27% of the variance across component predictors and 56% and 51% of the variance in elevated AMSKI risk (Table 3).

### 3.4. PLS-DA Components

**Figure 3:**
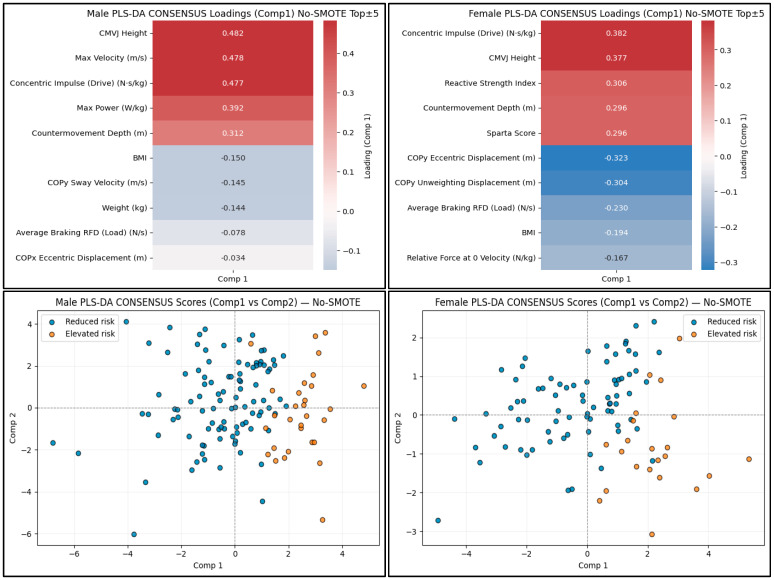
No-SMOTE PLS-DA consensus component loading heatmaps and scatterplots. The top 5 positive and negative male and female consensus loadings for the No-SMOTE PLS-DA Component 1 are shown (**top**). Scatterplots by sex of PLS-DA Component 1 and 2 (**bottom**), differentiated by AMSKI risk status.

The PLS-DA consensus estimates showed some differences between sexes in Component 1 loadings. In males, jump height, concentric impulse (Drive), maximum velocity, and maximum power had the largest positive loadings on component 1 (Figure 3). COPy sway velocity, BMI, and average braking RFD (Load) had the largest negative loadings. In females, concentric impulse (Drive), jump height, RSI, counter-movement depth, and the Sparta score had the most substantial positive loadings. At the same time, COPy eccentric displacement, COPy unweighting displacement, Load, and BMI had the most substantial negative loadings (Figure 3). Scatterplots of the first two PLS-DA components revealed substantial separation between Elevated and Reduced AMSKI risk participants, with elevated-risk individuals clustering at greater Component 1 scores (Figure 3). This pattern was consistent in both the male and female cohorts. The predictor sets and rank ordering were similar, albeit with some minor differences in the selected predictors between SMOTE and No-SMOTE models, supporting the robustness of the reported loading patterns to class-balancing strategy (Figure S7).

### 3.5. LASSO-Selected Predictors for CMVJ Height (PLSR Models)

LASSO bootstrap resampling retained 20 and 15 consensus predictors in males and females, respectively (Table 2; Figures S3 and S6). Non-retained predictors were primarily observed from timing-related, acceleration, and select COP displacement and sway domains in females, whereas fewer exclusions were observed in males and were largely confined to performance-derived force and some sway measures.

### 3.6. PLSR Consensus Model Performance and Components

**Figure 4:**
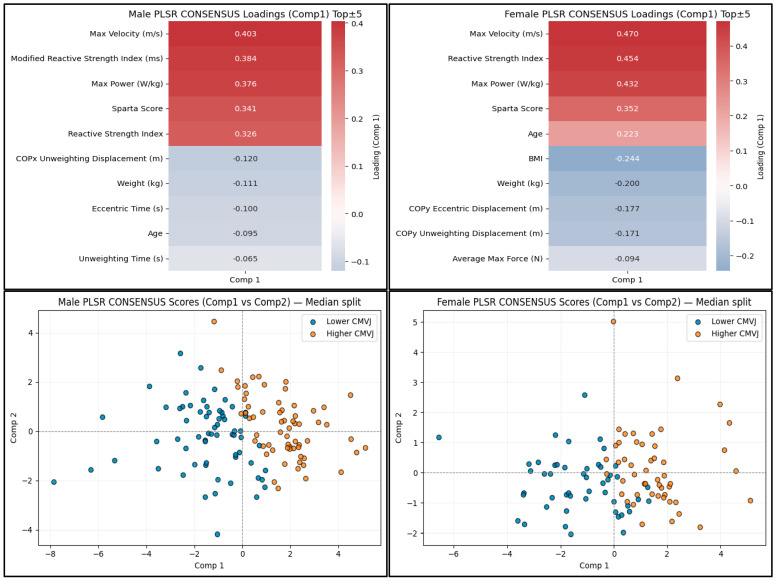
PLSR consensus component loading heatmaps and scatterplots. The top 5 positive and negative male and female consensus loadings for No-SMOTE PLSR Component 1 (**top**) are shown. Male and female scatterplots of PLSR Components 1 and 2 (**bottom**), differentiated by high or low jump height. The median was used as the cutoff to determine higher or lower CMVJ height.

The bootstrapped PLSR consensus models achieved excellent performance with an R^2^ = 0.96 (95% C.I. [0.90–0.98]; RMSE = 0.14, 95% C.I. [0.12–0.23]) in males and an R^2^ = 0.95 (95% C.I. [0.85–0.98]; RMSE = 0.11, 95% C.I. [0.08–0.17]) in females (Tables S1 and S3). In males, the top positive consensus loadings on Component 1 included maximum velocity, RSI/mRSI, maximum power, and the Sparta Score. The top negative loadings included COPx (mediolateral) unweighting displacement, body mass (weight in kgs), eccentric time, and age. In females, the top positive loadings on Component 1 included maximum velocity, RSI, maximum power, and the Sparta Score, whereas the top negative loadings included BMI, COPy eccentric, and COPy unweighting displacement. Scatterplots of Components 1 and 2 show substantial separation, with jump scores above the median clustering at greater Component 1 scores (Figure 4). The first two components explained 39% and 38% of the variance across the two component predictors and 96% and 95% of the variance in CMVJ height in males and females, respectively (Table 3).

### 3.7. Combined Importance Vector Analysis

**Figure 5:**
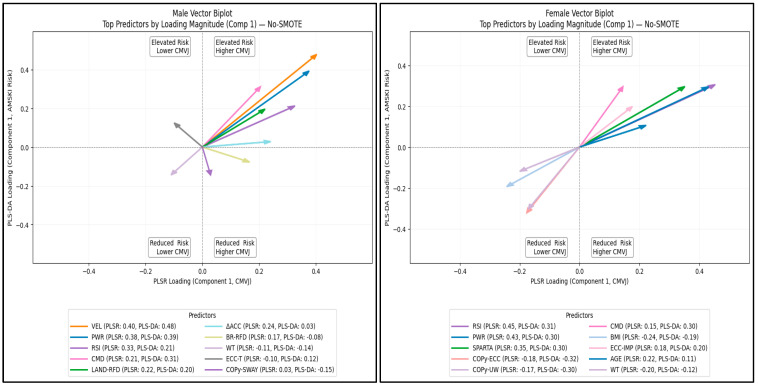
Consensus vector biplots for AMSKI risk vs. CMVJ height by sex (No-SMOTE). Each predictor is shown as a vector, using its Component 1 consensus loadings for elevated AMSKI risk and CMVJ height as coordinates. The vector’s direction indicates the pattern of association with each outcome, and its length (Euclidean norm) represents the combined importance.

Figure 5 shows the top 10 predictors, ranked by combined importance vector, and patterns of association for each outcome (AMSKI risk and CMVJ). In males, countermovement depth, maximum velocity, RSI, maximum power, landing rate of deceleration, and change in acceleration had a balanced association with higher CMVJ and elevated AMSKI risk. Load and COPy sway velocity were associated with reduced AMSKI risk and higher CMVJ, and body mass was associated with reduced AMSKI and lower CMVJ. Conversely, eccentric time was associated with elevated risk and lower CMVJ height. In females, countermovement depth, eccentric impulse, the Sparta score, maximum power, and RSI showed a balanced association with higher CMVJ and elevated AMSKI. However, BMI, COPy eccentric displacement, and COPy unweighting displacement had a more balanced association between reduced AMSKI risk and lower CMVJ. The vector biplots in the No-SMOTE condition did not vary substantially from the SMOTE condition (Figure S8).

### 3.8. Male Post Hoc Effects

Braking RFD (Load) exhibited a significant inverse association with AMSKI risk (β = −1.13, *p* = 0.002). Load was also positively associated with jump power (β = 0.15), which strongly predicted increased AMSKI risk, yielding a significant positive indirect effect (β = 0.46, 95% C.I. [0.03, 0.96]). The Load × power interaction was not significant.

COPy sway velocity demonstrated a significant inverse association with AMSKI risk (β = −0.72, *p* = 0.037). The indirect effect via power was positive but not statistically significant (β = 0.29, 95% C.I. [0.08, 0.72]). No significant interaction with power was observed.

BMI showed no significant direct (β = 0.47, *p* = 0.086) or interaction effects (β = −0.25, *p* = 0.25) with AMSKI risk. BMI was negatively associated with jump power (β = −0.29), and because power predicted increased risk, BMI produced a significant indirect effect (β = −0.66, 95% C.I. [−1.22, −0.22]).

The Sparta Score showed a nonsignificant direct association with injury risk (β = 0.75, *p* = 0.09) but was positively associated with jump power (β = 0.52), resulting in a significant positive indirect effect (β = 1.09, 95% C.I. [0.57, 1.83]). No significant moderation of the power–risk association was detected.

### 3.9. Female Post Hoc Results

COPy eccentric displacement showed no significant direct (β = −0.21, *p* = 0.53) or interaction effects (β = −0.27, *p* = 0.41). The indirect effect was also nonsignificant (β = −0.21, 95% C.I. [−0.51, 0.02]).

BMI demonstrated no significant direct (β = 0.11, *p* = 0.68) or interaction effects (β = 0.01, *p* = 0.96). BMI was negatively associated with jump power (β = −0.30), and this relation yielded a significant indirect effect (β = −0.34, 95% C.I. [−0.72, −0.04]).

Braking RFD (Load) exhibited a significant inverse association with AMSKI risk (β = −4.14, *p* < 0.001). A significant Load × power interaction was observed (β = 2.24, *p* = 0.008), though the indirect effect was not significant (β = 0.25, 95% C.I. [−0.12, 0.65]).

The Sparta Score demonstrated no significant direct effect (β = −0.44, *p* = 0.057) but was moderately associated with jump power (β = 0.46), producing a significant positive indirect effect (β = 0.61, 95% C.I. [0.26, 1.08]). The interaction with power was not statistically significant.

## 4. Discussion

Despite widespread adoption of commercial force plate technologies for athlete monitoring, the biomechanical features underlying musculoskeletal injury risk remain poorly understood. The emergence of force plate platforms incorporating proprietary injury risk classifications and other derived metrics complicates this issue. This lack of transparency limits the interpretability, clinical relevance, and translational utility of these proprietary risk metrics, particularly when the association between jump performance and injury risk contradicts established understanding. As such, the present study sought to clarify how force-time curve variables relate to CMVJ performance and AMSKI risk across sex.

In this investigation, we demonstrated a multistep method of filtering interrelated force-time curve metrics derived from the CMVJ test, using a commercial force plate, to ascertain the importance of these variables relating to CMVJ performance and algorithmically derived injury risk, as well as sex differences. We also examined the combined importance of force-time curve variables in predicting both jump performance and AMSKI risk. PLS analyses showed that a cluster of variables comprising increased jump height and other associated power metrics, along with lower COP sway velocity/displacement and braking rate of force development, explains substantial proportions of elevated AMSKI risk across sex. However, we observed several trade-offs between CMVJ height and AMSKI risk that were mediated by certain variables, which will be discussed below.

In this mixed-sex and sport cohort, a trade-off exists between jump height and AMSKI risk. This conflicts with a recent analysis that reported an association between increased AMSKI risk and lower CMVJ, performed within 3 months before injury [13]. However, a recent systematic review questioned the predictive value of jump performance measures on injury risk [8], finding that only 30% of studies using jump height measures showed an association, whereas 89% of the studies using kinetic and/or kinematic measures found associations with injury. Taken together, jump height or power alone might not be optimal for predicting injury risk.

The Sparta Score is calculated by factoring Load, Explode, and Drive and the balance among these measures [4], which assumes the importance of this metric on jump height and MKSI risk. Although our study confirmed a positive association between the Sparta score and jump height, the association with AMSKI risk was mediated by power. Few studies are available, although one study suggested limited predictive utility in using algorithmically derived composite scores [14]. In a retrospective military trainee cohort study, no significant associations were found between composite scores (Sparta Score and MSK Health score) and increased injury risk, despite excellent test–retest reliability [4]. Moreover, there were no significant associations between injury rate and vertical jump, or proprietary component scores, Load, Explode, and Drive. In contrast, a prospective study in a similar cohort found that one-unit increases in the MSK Health score increased the likelihood of injury by 4.7% [12]. However, no associations were found for the Sparta Score or Sparta’s AMSKI risk categories.

In contrast, a recent prospective study on the association between Sparta-derived metrics and ACL injury found significantly reduced Explode values and significantly increased Drive values among those who sustained an ACL injury during the follow-up period after the CMVJ test [15]. Furthermore, increased Load:Expode and reduced Explode:Drive ratios were found for those who sustained an ACL injury, suggesting that the bivariate relationships among Load, Explode, and Drive may be valid predictors of injury. There were no significant differences between the injured and non-injured groups for Load [15]. In contrast, we found significantly increased values for Drive and Explode in the male and female elevated-risk groups. We also found significantly reduced Load values in the female elevated-risk group.

In both males and females, reduced braking RFD (Load) was an independent predictor of AMSKI risk. Our findings are consistent with the mechanistic aspects of injury. Load (the average braking RFD) represents an athlete’s ability to adequately manage decelerative forces, which has implications for injury risk. Deficits in decelerative ability, as measured by ground reaction forces, may be indicative of neuromuscular fatigue or maladaptive control strategies, which expose the lower limbs to greater mechanical loads and heighten the risk of tissue failure [16]. Notably, we observed increased countermovement depth in males and females and increased eccentric time in males, as major contributors to the elevated AMSKI risk signatures. This may signal a distinct neuromuscular control strategy reflecting reliance on a greater range of motion to generate impulse, potentially compensating for force production or deficits in stretch–shorten cycle capacity [17]. This compensatory control strategy potentially increases injury risk as greater countermovement depth exposes individuals to greater hip and knee forces [17,18]. Evidence suggests that jump performances with reduced countermovement depth limit movement time and joint excursions, resulting in a more efficient reflexive output. However, reduced countermovement depth also improves mRSI via decreases in time-to-take-off, making up for losses in jump height [17].

Our findings support a recent analysis reporting a paradoxical relationship in which increases in vertical jump were associated with significantly decreased values for Sparta’s proprietary MSK Health score [14]. This contradicts the conventional understanding of the injury-protective effect of greater power and strength and further questions the validity of Sparta’s algorithmically derived injury risk metric [14]. Alternatively, increased AMSKI risk scores may be the result of the positive association between power and Load, with relatively greater Load values decreasing AMSKI risk.

The PLS analysis revealed that dynamic balance and postural control variables, COPy sway velocity in males and COPy eccentric/unweighting displacement in females, were negatively loaded on Component 1 for elevated AMSKI risk. At the same time, COPy eccentric/unweighting displacement in females, and COPx unweighting displacement in males, were negatively loaded on Component 1 for jump height. Evidence is mixed concerning the importance of postural sway in predicting injuries and vertical jump performance [19,20,21,22]. Some studies utilizing standing postural sway error or functional assessments like the Y-Balance Test show increased ankle and knee injury risk with increased balance errors or decreased Y-Balance scores [21,22,23,24]. In general, however, functional assessments have been shown to lack discriminatory power, owing to the lack of anthropometric, sport, force, kinematic, and workload measures [23]. The discrepancies between our findings and the extant literature might be due to differences in how postural sway is measured or due to the test’s sensitivity relative to the population of interest, the movement/sport context, or the region of injury [23].

Additionally, a recent scoping review suggested that differences in postural control and balance performance were attributable to sport-specific training adaptations, sport- and activity-specific experience, or overall physical activity rather than to neuromuscular proficiency alone [25]. For example, one study involving 51 professional and youth soccer players found significant moderate correlations between dynamic and static balance performance, as measured by COP displacement [26]. However, professional players exhibited a smaller sway area than players at lower levels of competition.

Other evidence suggests that phase-specific ground reaction force (GRF) characteristics during dynamic balance tasks are better at discriminating between increased and decreased injury risk groups. For instance, early-phase COP displacement during a dynamic balance test (shifting from a double-leg stance to a single-leg stance) was associated with an increased risk of non-contact ACL injury at 1-year follow-up, indicating an inability to overcome the initial perturbation caused by the dynamic task [27]. Similarly, players who scored 2 standard deviations (SDs) below the average in mediolateral stability in the early phase and 2 SDs above the average for horizontal GRF in the later phase of stabilization during drop-jump landings had a 4.4-fold increase in subsequent ankle sprain risk in elite soccer players [20]. On the other hand, in activity-specific contexts where large perturbation is commonplace, such as trampoline events, elite athletes displayed greater COP sway and speed, but time-series entropy was reduced compared to that of sub-elite athletes, suggesting more efficient motor control strategies [28]. Studies examining dynamic balance during sport-specific or high-perturbation tasks [20,25,26,27,28], in conjunction with our results, indicate that not all postural excursions signal risk-increasing instability. Greater COP excursions may also index a more adaptive strategy to dynamically stabilize or absorb loads during high-impact activities or may reflect context-specific experience.

Consistent with prior research, BMI demonstrated an inverse association with power, with a small-to-moderate effect in males and a moderate effect in females [29]. Although BMI attenuated the power–risk pathway, this effect should not be interpreted as a protective mechanism, due to the established link between BMI and increased injury risk [30,31]. The connection between BMI and increased injury risk is multifactorial, as greater BMI is often indicative of reduced fitness levels, greater susceptibility to injuries due to increased external loads, impaired neuromuscular coordination, and blunted adaptive responses [31]. Excess fat tissue may also contribute to reduced explosive output by negatively impacting the efficiency of the stretch–shorten cycle via increased “dead” mass, differences in viscoelastic properties between muscle and fat, and impaired muscle energy metabolism [32,33].

Interestingly, RSI and mRSI were significantly reduced in the reduced-risk group for both males and females. However, decreased RSI values are typically associated with increased risk of real-world injury, as reported in prospective analyses [34,35]. The conflict between our findings and those of previous studies may relate to differences in how RSI was derived. RSI is traditionally calculated from drop jumps, whereas mRSI (and CMVJ-derived RSI) reflects force-time characteristics of the CMVJ. Drop jumps expose the lower body to greater decelerative forces when compared to a standard vertical jump test, which may be a crucial aspect of the drop jump test’s validity. As a result, these indicators are not directly interchangeable across jump modalities and should be interpreted within the context of the movement from which they are derived. More research is needed to clarify the importance of the drop jump test relative to other methods for deriving RSI.

### Limitations

One of the main limitations of this study concerns the validity of using algorithmically derived injury risk as a proxy for actual injury risk. Although some relationships between force-time metrics and AMSKI risk align with known injury mechanisms, other conflicting results highlight the need for further research before recommending AMSKI risk metrics for practical use. For example, the paradoxical power–risk trade-off we observed might result from the injury-risk algorithm rather than from biomechanical factors. Consequently, we caution readers not to assume that power increases will necessarily lead to increased injury risk. Another important limitation was the lack of sport-specific analyses. Since injury risk varies depending on neuromuscular demands, loading patterns, and sport-specific injury factors (e.g., contact versus non-contact, playing surface, etc.), future research should further explore the connection between sport-specific force-time curve metrics and real-world injury incidence.

Although PLS is useful for identifying clusters of predictors that maximize covariance in outcomes, it is not designed for significance testing. However, the primary purpose of our study was to explore the importance of force-time curve variables, alongside proprietary metrics derived from a commercial force plate platform, which may help to inform clinical or training-related decision-making. Commercial force plate platforms generate a large number of interrelated output metrics, creating analytical challenges common to high-dimensional collinear datasets. Traditional regression approaches often address multicollinearity by removing correlated predictors to simplify predictive models, at the expense of biomechanical context. LASSO regression provides an alternative by performing variable selection. However, in the presence of strong collinearity, LASSO may arbitrarily retain one of several correlated predictors and exhibit instability in selected feature sets across resamples. To address this limitation, we employed bootstrap resampling of LASSO models, allowing predictor selection frequency to be quantified and thereby improving the stability and interpretability of the resulting consensus predictor sets.

## 5. Conclusions

This study sought to identify the multivariate jump-mechanics profiles most strongly linked to algorithmically derived musculoskeletal injury risk and CMVJ performance using a multi-stage analytic approach in which LASSO bootstrapping facilitated variable screening, and PLS provided the primary framework for interpreting the underlying structure of AMSKI risk. By vectorizing PLS component scores from both the CMVJ and AMSKI models, we were able to contextualize the relative importance of each biomechanical variable across outcomes. Collectively, the findings indicate that AMSKI risk is not driven by any single jump characteristic, but rather by the interplay among performance-, control-, and stability-related factors. Although some individual predictors showed no significant univariate differences between injury-risk groups, the multivariate analyses revealed meaningful conditional effects that only became apparent when accounting for the shared variance among correlated CMVJ variables.

Athletes exhibiting greater jump-performance outputs, particularly those with greater concentric power, velocity, and related explosive metrics, consistently demonstrated elevated AMSKI risk, suggesting that increased mechanical output may impose greater demands on musculoskeletal tissues or reflect movement strategies that increase vulnerability. In contrast, braking-control capacity, indexed by the Load variable, showed a strong inverse association with AMSKI risk despite its nonsignificant univariate group differences in males. This pattern emphasizes the protective value of eccentric control and force-modulation strategies, which may mitigate the risks associated with high-output movement profiles.

Taken together, these findings highlight the utility of multivariate modeling for capturing the complex biomechanical interactions that contribute to injury. Algorithmically derived injury risk appears to emerge from the balance, or imbalance, between an athlete’s performance capacity and their ability to modulate and decelerate the forces they generate. Athletes who achieve greater jump heights and power outputs may be at heightened risk if not paired with sufficient braking and deceleration control.

Overall, although CMVJ-derived performance and braking-control metrics offer practical insights for training and injury-risk management, coaches and clinicians should interpret them cautiously in light of ongoing questions about the ecological validity and generalizability of algorithmically derived composite scores. Prioritizing validated, mechanism-based measures remains essential when developing interventions aimed at enhancing performance while reducing injury risk.

## Figures and Tables

**Table 1:** Differences in Sparta force plate measures: elevated vs. reduced AMSKI risk. Values are standardized as z-scores within sex. Negative values are below the mean; positive values are above the mean. ^a^ Benjamin–Hochberg false discovery rate correction. * Significant at *p* < 0.01; ** Significant at *p* < 0.001.

Feature	Mean—Elevated Risk	SD	Mean—Reduced Risk	SD	*p*-Value (^a^ FDR-adj.)
Males					
CMVJ Height	1.495	0.496	0.349	0.720	<0.001 **
Concentric Impulse (Drive) (N·s/kg)	1.398	0.409	0.351	0.747	<0.001 **
Avg Relative Concentric Force (Explode) (N/kg)	0.855	0.647	0.336	0.918	0.009 *
Average Braking RFD (Load) (N/s)	0.076	0.766	0.498	0.932	0.090
Reactive Strength Index	1.061	0.867	0.383	0.912	0.001 *
Modified Reactive Strength Index (mRSI)	1.080	0.818	0.401	0.878	0.001 *
Sparta Score	0.841	0.921	−0.153	1.168	<0.001 **
Females					
CMVJ Height	−0.389	0.518	−0.925	0.432	<0.001 **
Concentric Impulse (Drive) (N·s/kg)	−0.292	0.535	−0.915	0.557	<0.001 **
Avg Relative Concentric Force (Explode) (N/kg)	−0.604	1.013	−0.596	0.746	0.556
Average Braking RFD (Load) (N/s)	−0.984	0.553	−0.392	0.895	<0.001 **
Reactive Strength Index	−0.338	0.652	−0.812	0.410	0.001 *
Modified Reactive Strength Index (ms)	−0.433	0.677	−0.815	0.456	0.010 *
Sparta Score	−0.054	0.577	−0.119	0.694	0.980

**Table 2:** Consensus predictors (selection frequency ≥ 40%) retained in sex-specific PLSR models predicting CMVJ height and PLS-DA models classifying AMSKI risk using the observed (No-SMOTE) class distribution. ✓ indicates the predictor was retained in the model.

Category	Predictor	PLSR Female	PLSR Male	PLS-DA Female (No-SMOTE)	PLS-DA Male (No-SMOTE)
Performance	CMVJ Height			✓	✓
	Max Velocity (m/s)	✓	✓		✓
	Max Power (W/kg)	✓	✓	✓	✓
	Reactive Strength Index	✓	✓	✓	✓
	Modified Reactive Strength Index (ms)		✓		
	Concentric Impulse (Drive) (N·s/kg)			✓	✓
	Relative Force at 0 Velocity (N/kg)			✓	✓
Kinetics	Average Max Force (N)	✓	✓	✓	✓
	Eccentric Impulse (N·s/kg)	✓	✓	✓	✓
Acceleration	Jump Max Δ Acceleration (m/s^3^)		✓	✓	✓
	Jump Max Acceleration (m/s^2^)		✓		
Control/Braking	Avg Landing Rate of Deceleration (m/s^3^)	✓	✓	✓	✓
	Average Braking RFD (Load) (N/s)		✓	✓	✓
Control/Timing	Unweighting Time (s)		✓		
	Concentric Time (s)			✓	✓
	Eccentric Time (s)		✓	✓	✓
	Time to Take-Off (s)			✓	✓
COP	COPx Eccentric Displacement (m)	✓	✓	✓	✓
	COPy Eccentric Displacement (m)	✓	✓	✓	✓
	COPx Unweighting Displacement (m)		✓	✓	✓
	COPy Unweighting Displacement (m)	✓		✓	
COP/Sway	COPx Sway Velocity (m/s)	✓			✓
	COPy Sway Velocity (m/s)		✓		✓
Anthropometric/Demographic	Age	✓	✓	✓	✓
	Body mass (kg)	✓	✓	✓	✓
	Height (in)			✓	
	BMI	✓		✓	✓
Composite	Sparta Score	✓	✓	✓	
Technique/Strategy	Countermovement Depth (m)	✓	✓	✓	✓

**Table 3:** Consensus PLS models—variance explained.

Group	Component	Component Variance Explained	Component Cumulative Variance Explained	Outcome Cumulative Variance Explained (R^2^)
AMSKI Elevated Risk (No-SMOTE)				
Male	1	0.17	0.17	0.48
	2	0.21	0.38	0.56
Female	1	0.18	0.18	0.31
	2	0.09	0.27	0.51
CMVJ Height				
Male	1	0.28	0.28	0.88
	2	0.11	0.39	0.96
Female	1	0.27	0.27	0.87
	2	0.11	0.38	0.95

## Data Availability

The raw data supporting the conclusions of this article will be made available by the authors on request.

## Supplementary Materials

The following supporting information can be downloaded at: https://www.mdpi.com/article/10.3390/sports14020067/s1, Figure S1: Consensus predictors for the PLS-DA SMOTE model in males; Figure S2: Consensus predictors for the PLS-DA No-SMOTE Model in males; Figure S3: Consensus predictors for the PLSR Model in males; Figure S4: Consensus predictors for the PLS-DA SMOTE model in females; Figure S5: Consensus predictors for the PLS-DA No-SMOTE Model in females; Figure S6: Consensus predictors for the PLSR model in females; Figure S7: SMOTE PLA-DA Consensus Component Loading Heatmaps and Scatterplots; Figure S8: Each predictor is shown as a vector, using its Component 1 loadings for AMSKI risk under SMOTE and CMVJ height as coordinates; Table S1: PLSR Consensus Model Performance; Table S2: PLS-DA Consensus Model Performance; Table S3: PLSR Bootstrap Performance (95% Confidence Intervals); Table S4: PLSDA Bootstrap (N = 500) Performance Differences: SMOTE vs. No-SMOTE (Males); Table S5: PLSDA Bootstrap (N = 500) Performance Differences: SMOTE vs. No-SMOTE (Females).

## Abbreviations

The following abbreviations are used in this manuscript:

CMVJ height	The maximal vertical displacement achieved during the countermovement jump	in
Jump maximum change in acceleration	The greatest instantaneous change in vertical acceleration recorded across the jump	m/s^3^
Eccentric impulse	The total impulse generated during the eccentric (downward) phase of the jump, normalized to body mass	Ns/kg
Concentric impulse	The total impulse generated during the concentric (upward) phase, normalized to body mass	Ns/kg; “Drive”
Maximum velocity	The peak vertical velocity attained during the concentric phase	m/s
Average maximum force	The mean of the maximal vertical ground reaction forces recorded across the jump	N
Maximum power	The highest instantaneous power output generated during the concentric phase, normalized to body mass	W/kg
Unweighting time	The duration between movement initiation and the minimum force point that precedes downward acceleration	s
Eccentric time	The total duration of the eccentric phase	s
Concentric time	The total duration of the concentric phase	s
Time to take-off	The elapsed time from movement onset to departure from the force plate	s
Time to maximum acceleration	The time from movement initiation to the peak vertical acceleration	s
Countermovement depth	The maximum downward displacement of the center of mass during the eccentric phase	m
Average braking rate of force development	The mean rate at which force increases during the eccentric braking phase	N/s; “Load”
Average relative concentric force	The average concentric force output normalized to body mass	N/kg; “Explode”
COPx unweighting displacement	Mediolateral center-of-pressure displacement during the unweighting phase	m
COPy unweighting displacement	Anteroposterior center-of-pressure displacement during the unweighting phase	m
COPx eccentric displacement	Mediolateral center-of-pressure displacement during the eccentric phase	m
COPy eccentric displacement	Anteroposterior center-of-pressure displacement during the eccentric phase	m
COPx sway velocity	The mean mediolateral sway velocity across the jump phases	m/s
COPy sway velocity	The mean anteroposterior sway velocity across the jump phases	m/s
Average landing rate of deceleration	The mean rate at which vertical velocity decreases during landing	m/s^3^
Relative force at zero velocity	The concentric force output at the instant center-of-mass velocity passes through zero	N/kg
Modified Reactive Strength Index (mRSI)	A measure of explosive reactive ability calculated as jump height divided by ground-contact time	
Reactive Strength Index (RSI)	A measure of plyometric performance calculated as flight time divided by ground-contact time	
Sparta Score	A composite score integrating Load, Explode, and Drive variables derived from Sparta’s proprietary force-plate algorithm	
