# Supplementary material for: Sex-Specific Patterns of Force Plate-Derived Predictors for Vertical Jump Performance and Algorithmic Musculoskeletal Injury Risk in College Athletes
Source: Sports (Basel). 2026 Feb 5;14(2):67. doi: 10.3390/sports14020067 (PMC12944276; doi:10.3390/sports14020067)
Supplement: Supplementary file 1 [file sports-14-00067-s001.zip › sports-4076768-supplementary.pdf]

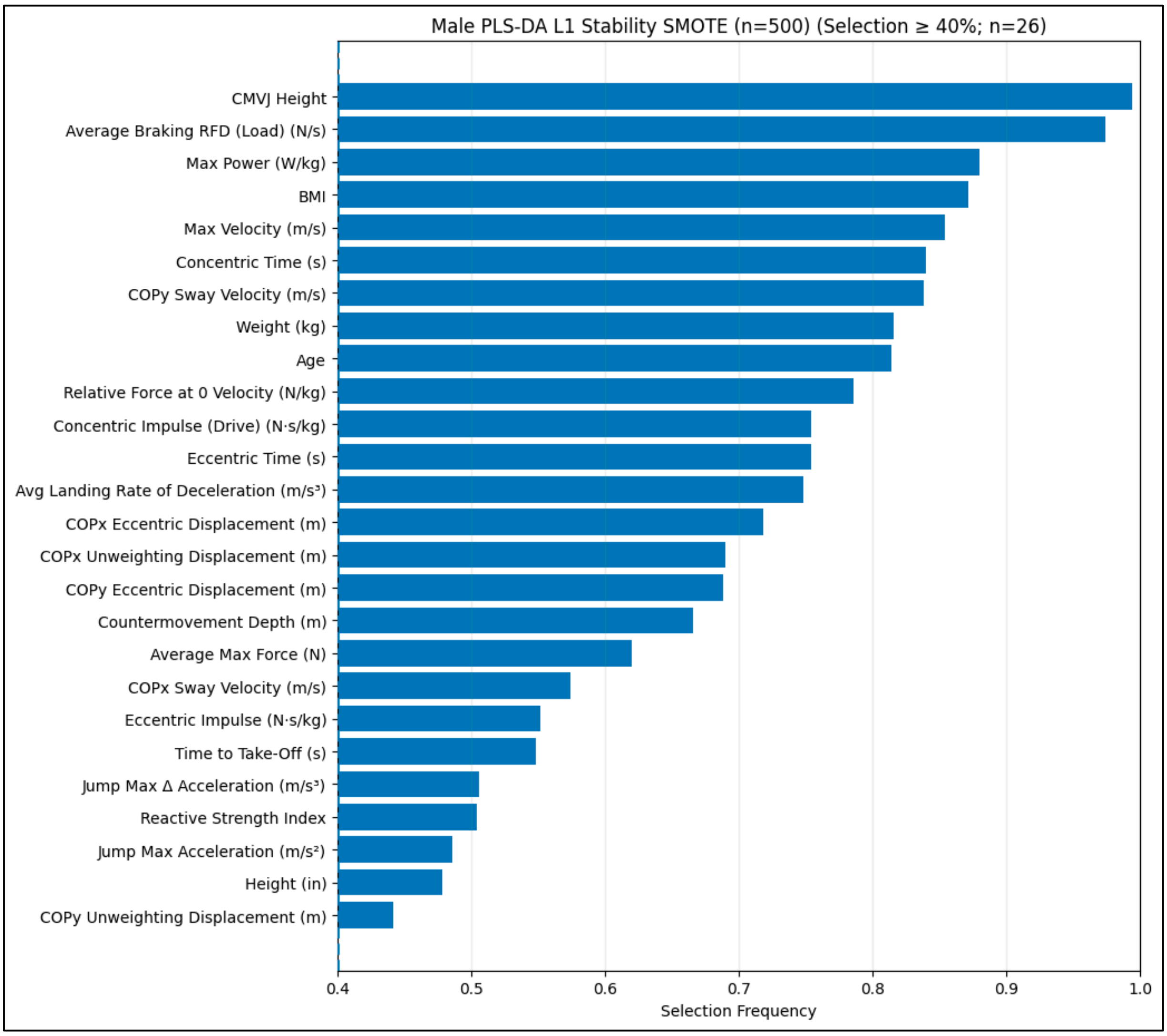

Figure S1. Consensus predictors for the PLS-DA SMOTE model in males. The proportion of bootstrap samples in which the SMOTE LASSO model retained each predictor is displayed. Predictors meeting or exceeding 40% were used in the PLS-DA SMOTE model.

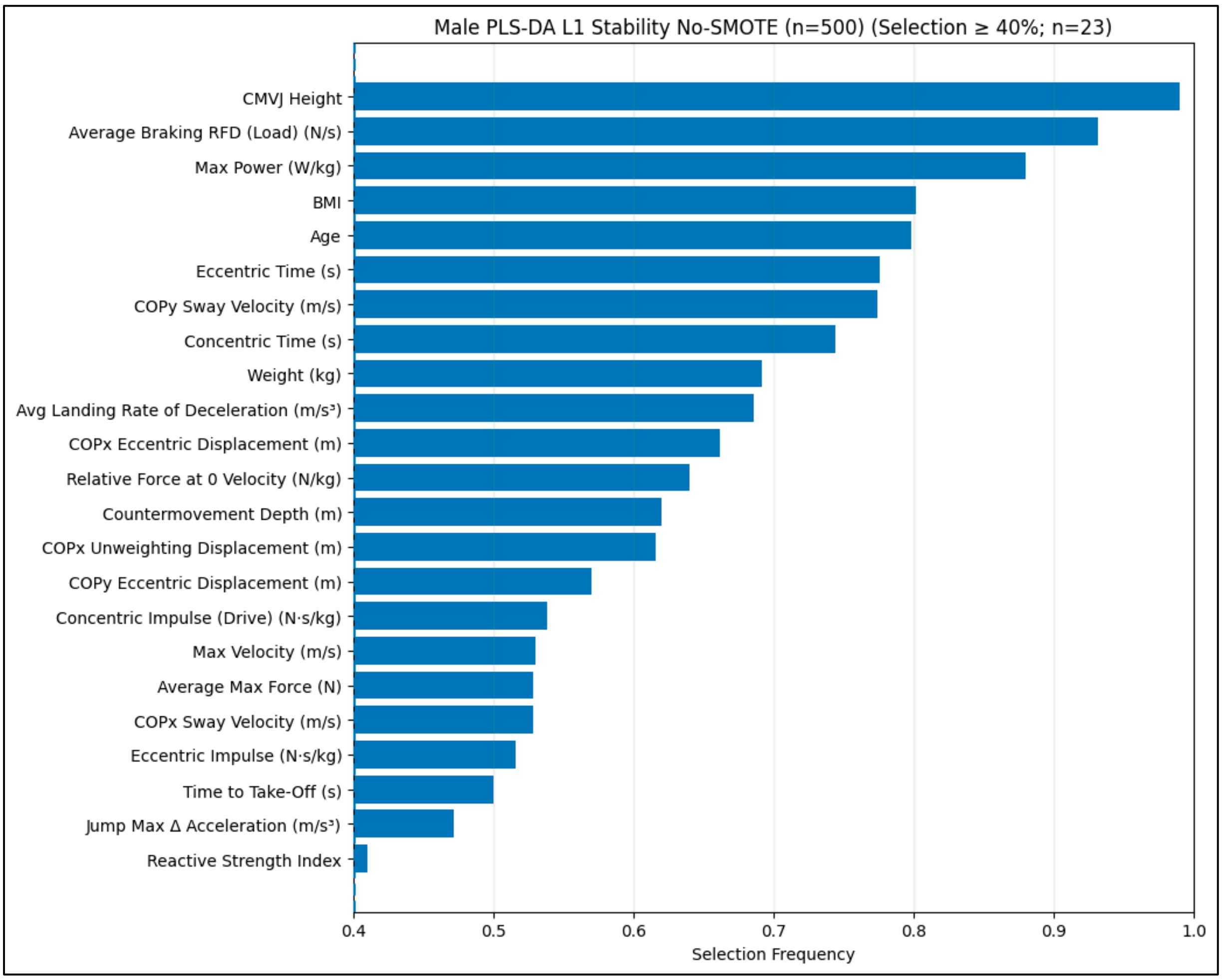

Figure S2. Consensus predictors for the PLS-DA No-SMOTE Model in males. The proportion of bootstrap samples in which the No-SMOTE LASSO model retained each predictor is displayed. Predictors meeting or exceeding 40% were used in the PLS-DA No-SMOTE model.

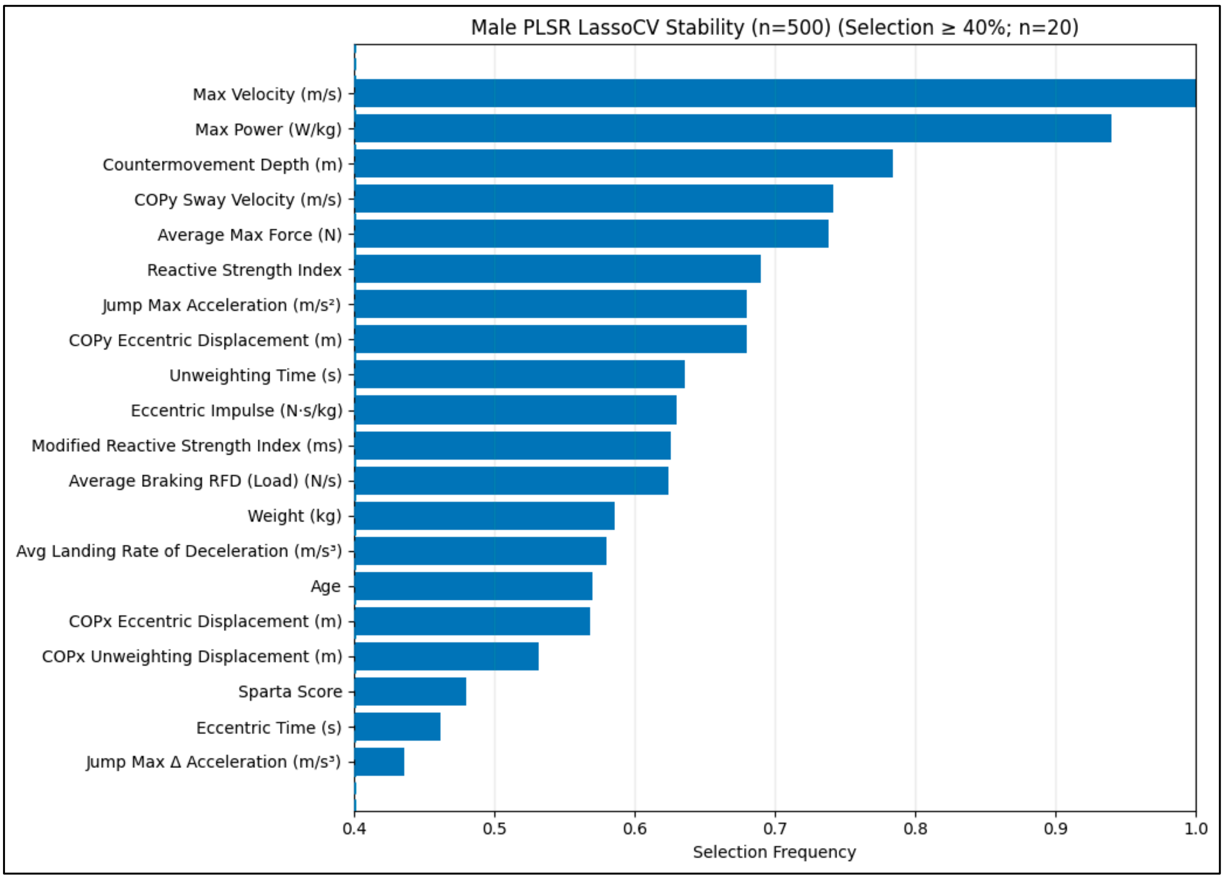

Figure S3. Consensus predictors for the PLSR Model in males. The proportion of bootstrap samples in which the sparse LASSO model retained each predictor is displayed. Predictors meeting or exceeding 40% were used in the PLSR model.

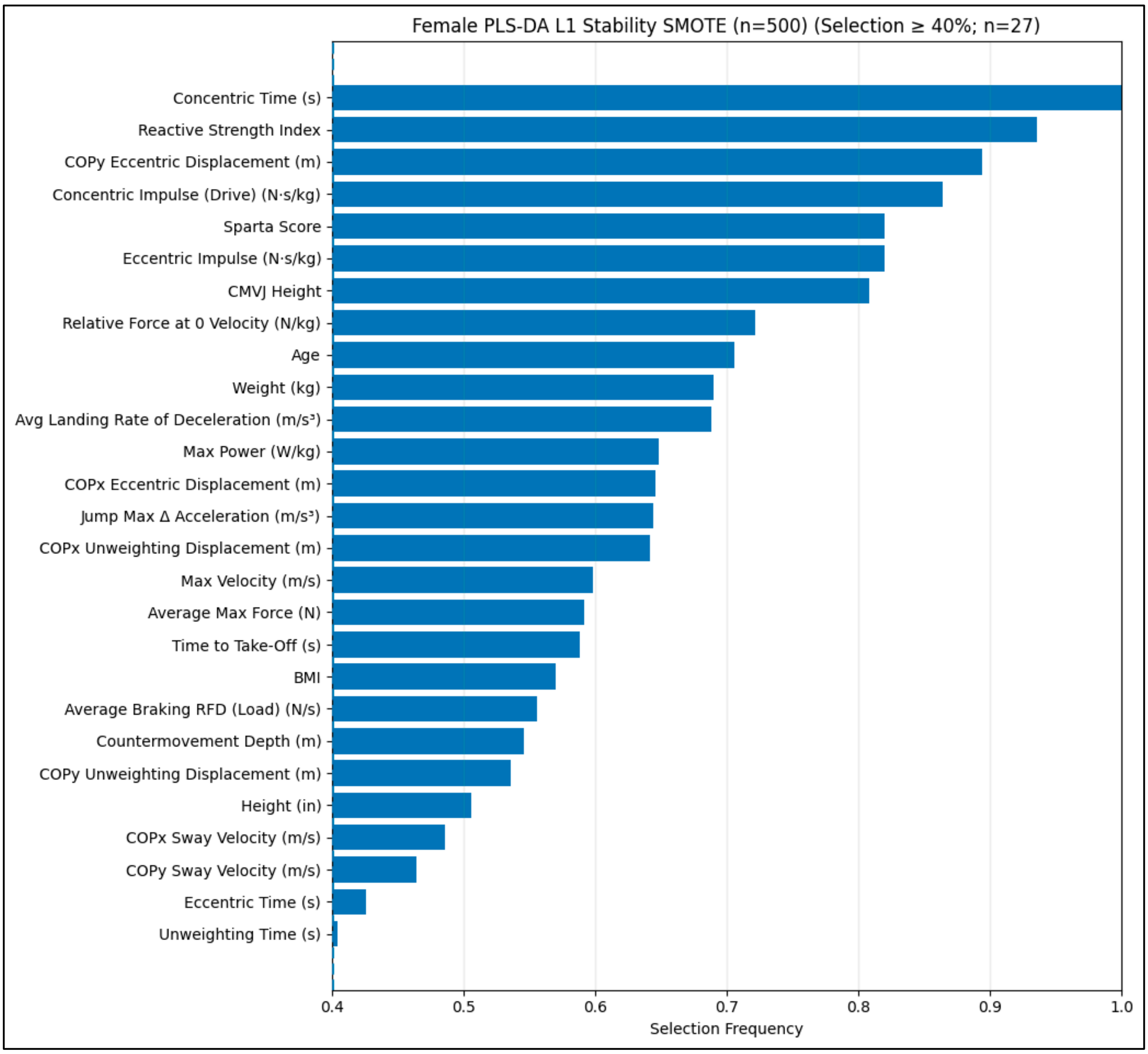

Figure S4. Consensus predictors for the PLS-DA SMOTE model in females. The proportion of bootstrap samples in which the sparse SMOTE LASSO model retained each predictor is displayed. Predictors meeting or exceeding 40% were used in the PLS-DA SMOTE model.

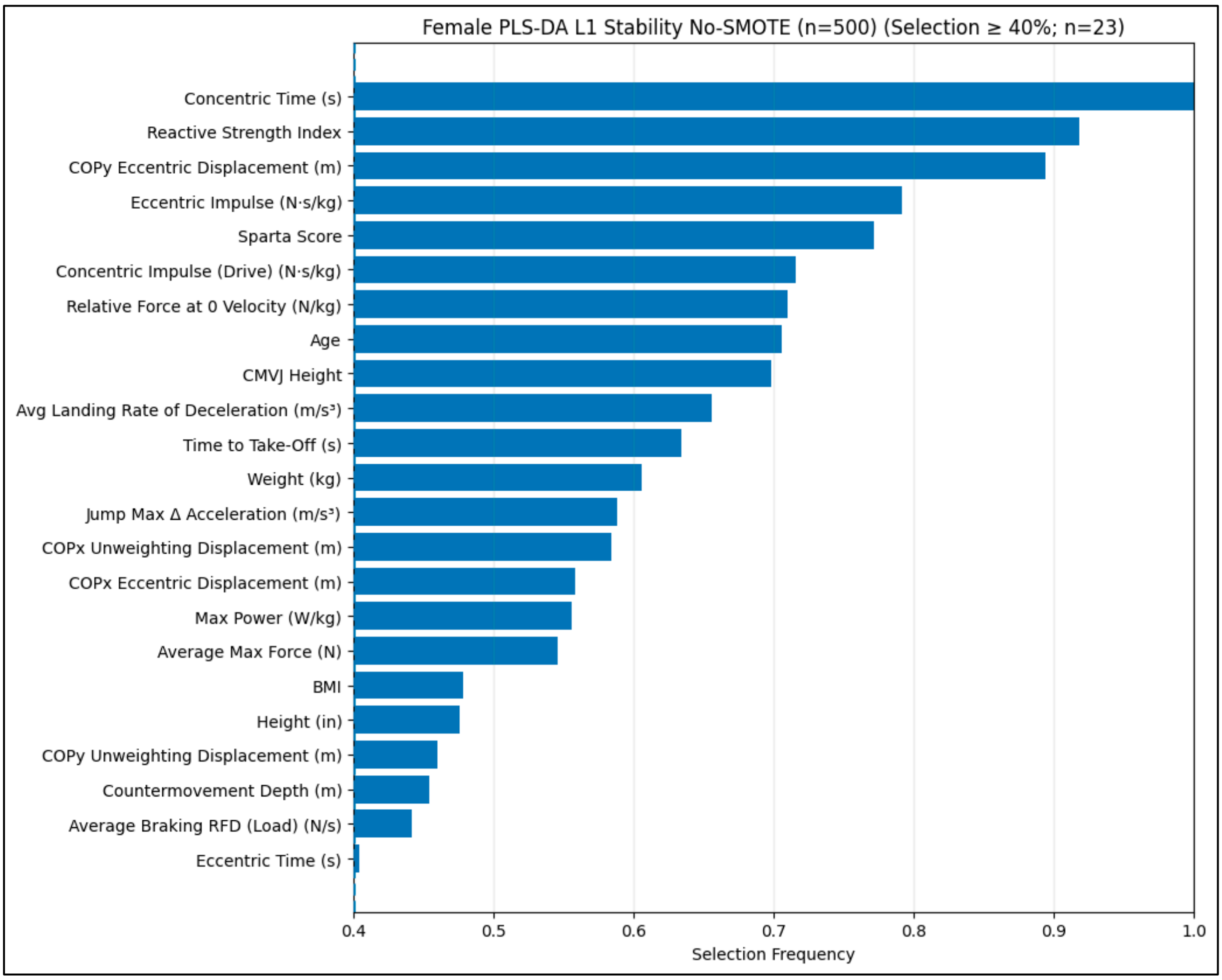

Figure S5. Consensus predictors for the PLS-DA No-SMOTE Model in females**.** The proportion of bootstrap samples in which the sparse No-SMOTE LASSO model retained each predictor is displayed. Predictors meeting or exceeding 40% were used in the PLS-DA No-SMOTE model.

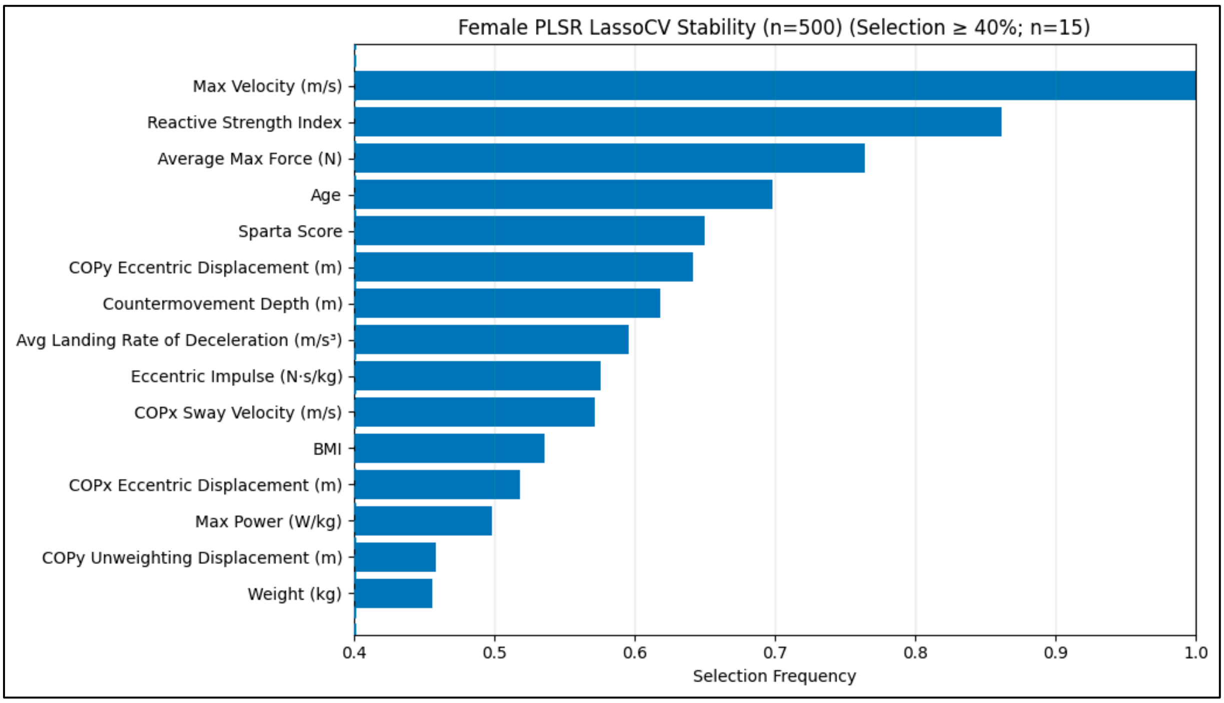

Figure S6. Consensus predictors for the PLSR model in females. The proportion of bootstrap samples in which the sparse LASSO model retained each predictor is displayed. Predictors meeting or exceeding 40% were used in the PLSR model.

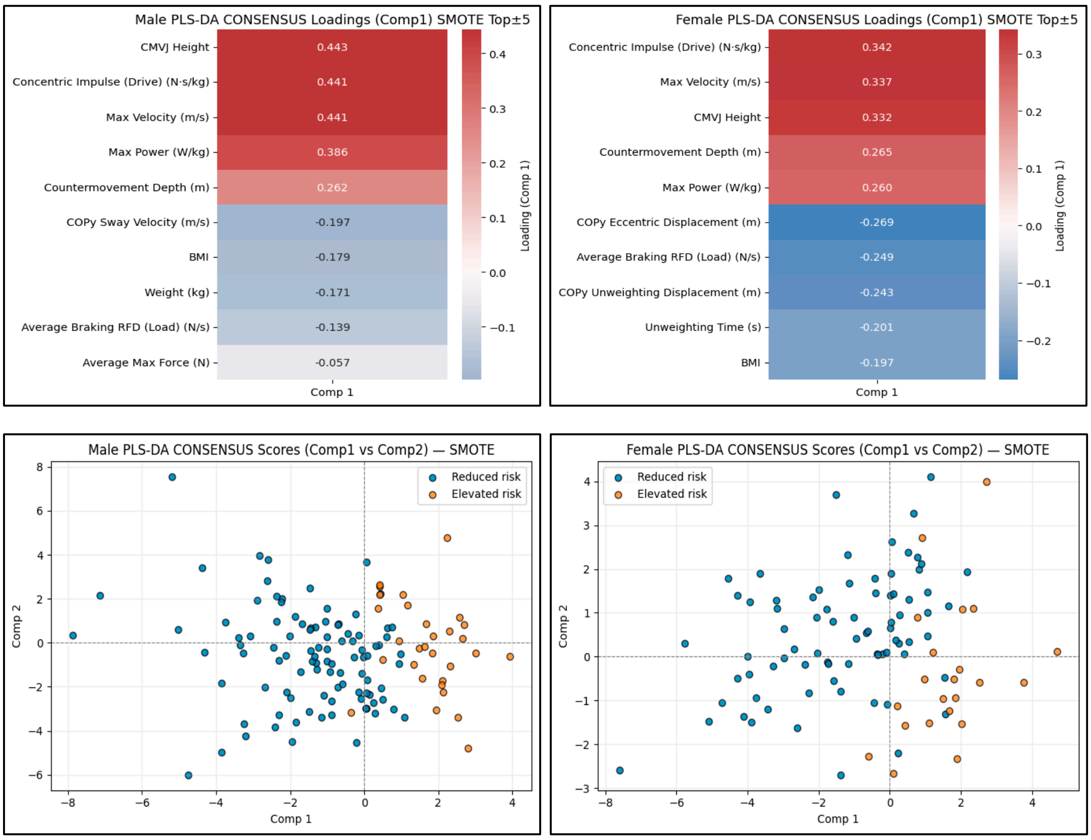

Figure S7. SMOTE PLA-DA Consensus Component Loading Heatmaps and Scatterplots.

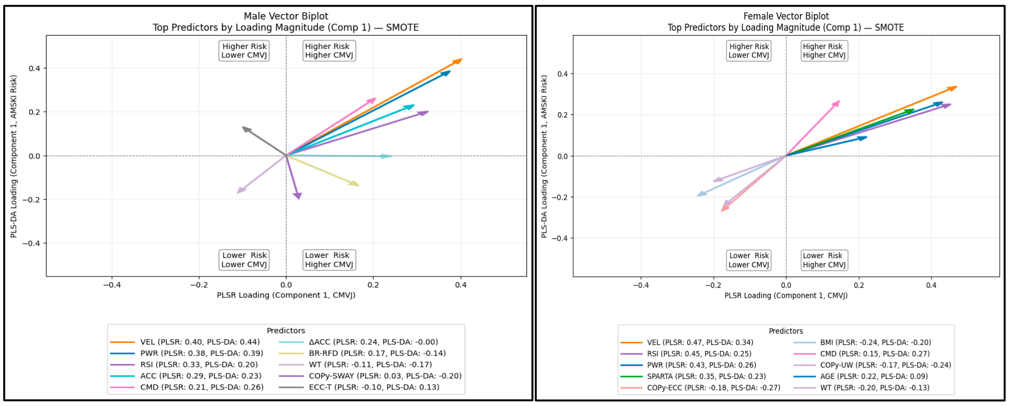

Figure S8. Each predictor is shown as a vector, using its Component 1 loadings for AMSKI risk under SMOTE and CMVJ height as coordinates. The vector's direction indicates the pattern of association with each outcome, and its length (Euclidean norm) represents the combined importance.

Table S1. PLSR Consensus Model Performance

Sex	R^2	RMSE
Male	0.9600	0.1395
Female	0.9545	0.1064

Table S2. PLS-DA Consensus Model Performance

Sex	Model	AUC	PR-AUC	Accuracy	Precision	F1
Male	SMOTE	0.9074	0.8680	0.7949	0.5333	0.6667
Male	No-SMOTE	0.8889	0.8039	0.8974	0.7778	0.7778
Female	SMOTE	0.9814	0.9379	0.9000	0.7000	0.8235
Female	No-SMOTE	0.9565	0.8806	0.8667	0.8000	0.6667

Table S3. PLSR Bootstrap Performance (95% Confidence Intervals)

Sex	R^2 Mean	R^2 SD	R^2 95% CI	RMSE Mean	RMSE SD	RMSE 95% CI
Male	0.9523	0.0185	0.9040 – 0.9779	0.1748	0.0279	0.1245 – 0.2335
Female	0.9320	0.0325	0.8519 – 0.9760	0.1209	0.0223	0.0809 – 0.1674

Table S4. PLSDA Bootstrap (N = 500) Performance Differences: SMOTE vs. No-SMOTE (Males)

Metric	Mean Δ	SD	Median	2.5%	97.5%
AUC	0.0042	0.0581	0.0032	−0.1171	0.1107
PR-AUC	0.0124	0.1218	0.0117	−0.2712	0.2535
F1	0.0351	0.1769	0.0198	−0.2826	0.4108
Accuracy	−0.0241	0.0760	−0.0256	−0.1795	0.1160
Precision	−0.1878	0.1990	−0.1905	−0.5455	0.1818

Table S5. PLSDA Bootstrap (N = 500) Performance Differences: SMOTE vs. No-SMOTE (Females)

Metric	Mean Δ	SD	Median	2.5%	97.5%
AUC	−0.0047	0.0920	−0.0062	−0.1750	0.1882
PR-AUC	−0.0139	0.1607	−0.0120	−0.3249	0.3007
F1	0.0261	0.2087	0.0000	−0.3458	0.4773
Accuracy	−0.0382	0.0849	−0.0333	−0.2333	0.1333
Precision	−0.1965	0.2544	−0.2102	−0.6000	0.3397
